# Examination of the changes in lower extremities related to progression of adult spinal deformity: a longitudinal study of over 22 years

**DOI:** 10.1038/s41598-020-68573-3

**Published:** 2020-07-14

**Authors:** Mutsuya Shimizu, Tetsuya Kobayashi, Hisashi Chiba, Issei Senoo, Satomi Abe, Keisuke Matsukura, Hiroshi Ito

**Affiliations:** 1 Department of Orthopaedic Surgery, Asahikawa Medical University, 2-1E Midorigaoka, Asahikawa, Hokkaido 0788510 Japan; 2 Furano Geriatric Health Services Facility, Furano, Japan

**Keywords:** Anatomy, Medical research

## Abstract

This longitudinal observational study investigated the relationship between changes in spinal sagittal alignment and changes in lower extremity coronal alignment. A total of 58 female volunteers who visited our institution at least twice during the 1992 to 1997 and 2015 to 2019 periods were investigated. We reviewed whole-spine radiographs and lower extremity radiographs and measured standard spinal sagittal parameters including pelvic incidence [PI], lumbar lordosis [LL], pelvic tilt [PT], sacral slope [SS] and sagittal vertical axis [SVA], and coronal lower extremity parameters including femorotibial angle (FTA), hip–knee–ankle angle (HKA), mechanical lateral distal femoral angle (mLDFA), mechanical medial proximal tibial angle (mMPTA) and mechanical lateral distal tibial angle (mLDTA). Lumbar spondylosis and knee osteoarthritis were assessed using the Kellgren–Lawrence (KL) grading system at baseline and at final follow-up. We investigated the correlation between changes in spinal sagittal alignment and lower extremity alignment and changes in lumbar spondylosis. The mean age [standard deviation (SD)] was 48.3 (6.3) years at first visit and 70.2 (6.3) years at final follow-up. There was a correlation between changes in PI-LL and FTA (R = 0.449, P < 0.001) and between PI-LL and HKA (R = 0.412, P = 0.001). There was a correlation between changes in lumbar spondylosis at L3/4 (R = 0.383, P = 0.004) and L4/5 (R = 0.333, P = 0.012) and the knee joints. Changes in lumbar spondylosis at L3/4 and L4/5 were related to changes in KOA. Successful management of ASD must include evaluation of the state of lower extremity alignment, not only in the sagittal phase, but also the coronal phase.

## Introduction

In recent years, with the aging population, the number of patients presenting to consultation rooms with both knee osteoarthritis (KOA) and lumbar degenerative diseases has increased^1^. In adult patients with spinal deformity, spinal kyphosis changes are caused by disc degeneration and vertebral fracture. A previous study reported that spinal deformity in the sagittal phase is progressive, and influences pain, risk of falls, and health-related quality of life (HRQOL)^2–5^.

Barry et al. reported the compensatory mechanisms associated with adult spinal deformity, and reported that in advanced spinal deformity, the body is affected from head to toe, including changes in hip extension and knee flexion in the sagittal phase to maintain spinal alignment^6^. On the other hand, KOA, which causes knee pain, knee contracture, decreased muscle strength, and decreased QOL, induced knee flexion in the sagittal phase and varus deformity in the coronal phase in the lower extremities, which is one of the characteristic changes proceeding KOA^7–9^. Both the sagittal phase and the coronal phase are very important in the evaluation of KOA.

Knee–spine syndrome has been previously reported by Murata et al. and Itoi who indicated that deformity of the knee joint influences spine deformity^10,11^. However, their studies did not examine the coronal phase of the lower extremities using X-ray. Because the hip joint exists between the spine and the knee joints, three-dimensional changes can occur in the lower extremities. It has been suggested that changes in the sagittal spine affect the coronal plane of the lower extremities; however, there are few reports on the relationship between the sagittal spine and coronal lower extremities.

The purpose of this study was to investigate the relationship between changes in spinal sagittal alignment (SSA) and changes in the coronal alignment of the lower extremities using a longitudinal cohort study of over 22 years.

## Material and methods

This study was conducted according to the STROBE guidelines for cohort studies. This study was a component of our ongoing prospective cohort study (ASAP study), which recruited community-dwelling volunteers from a population register.

**Figure 1:**
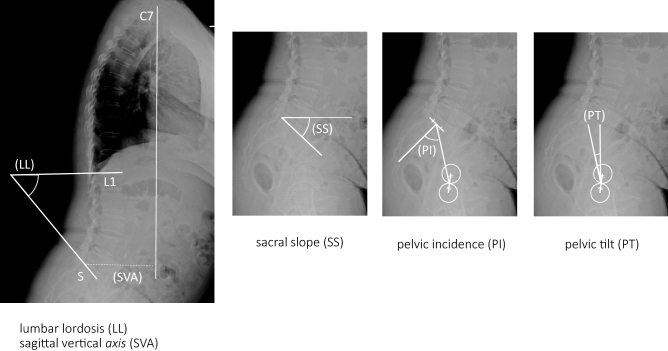
Radiographic measurements of spinal sagittal parameters. Image shows the lumbar lordosis (LL) which is the angle between the upper endplate of L1 and S1, the pelvic tilt (PT) which is the angle between the line drawn through the center of the femoral head and the midpoint of the sacral plate and the vertical reference, the pelvic incidence (PI) which is the angle between the line through the center of the femoral head and the midpoint of the sacral plate and the line perpendicular to the sacral plate, the sacral slope (SS) which is the angle between the horizontal line and upper endplate of S1, and the sagittal vertical axis (SVA) which is the distance between the C7 plumb line and the posterosuperior aspect of S1.

**Figure 2:**
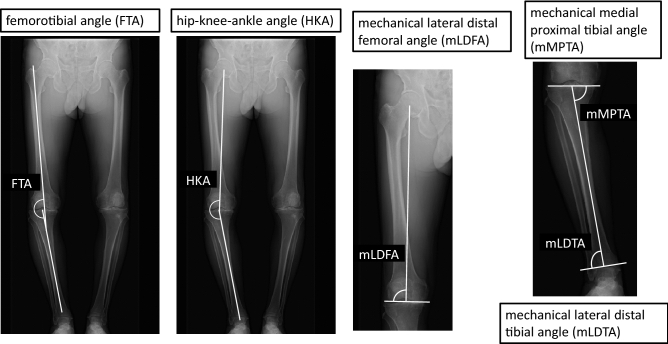
Radiographic measurements of coronal lower extremity parameters. Image shows the femorotibial angle (FTA) which is the lateral angle at the intersection between the femoral bone axis and the tibial bone, the hip–knee–ankle angle (HKA) which is the lateral angle between the mechanical axis of the femur and the tibia), the mechanical lateral distal femoral angle (mLDFA) which is the lateral angle between the mechanical axis of the femur and the distal femur joint line, the mechanical medial proximal tibia angle (mMPTA) which is the medial angle between the mechanical axis of the tibia and proximal tibia joint line, and the mechanical lateral distal tibia angle (mLDTA) which is the angle between the tibial mechanical axis and distal tibial joint surface.

A total of 58 female volunteers were included after they met the following criteria: (1) no history of hip and knee joint replacement surgery or spinal surgery, (2) no neuromuscular disease, and (3) attendance at appointments at least twice during the 1992–1997 and 2015–2019 periods. We reviewed whole-spine radiographs and lower extremity radiographs taken with the volunteers in a standing position. We measured standard spinal sagittal parameters including pelvic incidence [PI], lumbar lordosis [LL], pelvic tilt [PT], sacral slope [SS] and sagittal vertical axis [SVA] (Fig. 1). We also measured coronal lower extremity parameters including femorotibial angle [FTA], hip–knee–ankle angle [HKA], mechanical lateral distal femoral angle [mLDFA], mechanical medial proximal tibial angle [mMPTA] and mechanical lateral distal tibial angle [mLDTA] (Fig. 2). The parameters were defined as follows. The lumbar lordosis (LL) was the angle between the upper endplate of L1 and S1, pelvic tilt (PT) was the angle between the line drawn through the center of the femoral head and the midpoint of the sacral plate and the vertical reference, the pelvic incidence (PI) was the angle between the line through the center of the femoral head and the midpoint of the sacral plate and the line perpendicular to the sacral plate, the sacral slope (SS) was the angle between the horizontal line and upper endplate of S1, the sagittal vertical axis (SVA) was the distance between the C7 plumb line and the posterosuperior aspect of S1, the femorotibial angle (FTA) was the lateral angle at the intersection between the femoral bone axis and the tibial bone, the hip–knee–ankle angle (HKA) was the lateral angle between the mechanical axis of the femur and the tibia, the mechanical lateral distal femoral angle (mLDFA) was the lateral angle between the mechanical axis of the femur and the distal femur joint line, the mechanical medial proximal tibia angle (mMPTA) was the medial angle between the mechanical axis of the tibia and proximal tibia joint line, and the mechanical lateral distal tibia angle (mLDTA) was the angle between the tibial mechanical axis and distal tibial joint surface. The radiographic severity of osteoarthritis in the lumbar spine and knee joint was determined according to the Kellgren–Lawrence (KL) grading system, which is the most widely-used grading system. It classifies osteoarthritis into five grade scales (0–4) as follows. KL0 is normal, KL1 shows slight osteophytes, KL2 shows definite osteophytes, KL3 shows joint or intervertebral space narrowing with large osteophytes, and KL4 shows bone sclerosis, joint or intervertebral space narrowing, and large osteophytes. In our study, if a volunteer showed joint or intervertebral space narrowing without large osteophytes, the classification was KL3.

We investigated that the correlation between longitudinal changes in SSA and lower extremity alignment, changes grade of lumbar spondylosis at each level, and changes in the grade of KOA.

### Reliability test

Fourteen of 53 subjects were randomly selected to determine inter- and intra-rater reliability of measurement of the parameters; 6 spinal parameters were measured twice by 2 spine surgeons, with each set of measurements taken at least 2 weeks apart. Intra-class correlation coefficients (ICCs) were used to calculate inter- and intra-observer reliability. The calculated ICCs(r) were interpreted according to convention, with values of 0.90–1.0, 0.70–0.89, 0.50–0.69, 0.25–0.49, and < 0.24 representing excellent, good, fair/moderate, low, and poor agreement, respectively.

Asahikawa Medical University institutional review board approved utilization and publication of the study data, and all methods were performed in accordance with the relevant guidelines and regulations [Approval Number: 372].

Written informed consent was obtained from all volunteers after the purpose of our research was explained. The datasets generated during and/or analyzed during the current study are available from the corresponding author on reasonable request.

### Statistical analysis

All statistical analyses were performed using SPSS Statistics version 12 (IBM, Tokyo, Japan). All data were expressed as mean and standard deviation (SD). Normal distribution of the variables was verified using the Shapiro–Wilk test. To compare variables at baseline and final follow-up, if data were normally distributed, a *t* test was performed. If the lack of normality was significant, the Wilcoxon signed rank test was performed.

As measurement data were non-normal continuous variables, Spearman rank correlations were used to evaluate the associations between changes in spino-pelvic radiographic parameters and changes in coronal lower extremity radiographic parameters, and changes in lumbar spondylosis and changes in the grading of KOA.

P < 0.05 indicated a statistically significant difference.

Sub-analyses were performed to evaluate the association between changes in PI-LL and changes in FTA. Volunteers were divided into two groups based on changes in PI-LL: volunteers with a change of ≤ 10° (small change group: S Group) and volunteers with a change of > 10° (large change group: L Group). Between-group differences in measured parameters were evaluated using a Mann–Whitney analysis.

### Ethical approval

All procedures performed in studies involving human participants were in accordance with the ethical standards of the institutional and/or national research committee (approval no. 372) and with the 1964 Helsinki declaration and its later amendments or comparable ethical standards.

## Results

All volunteers were female with an average age of 47.4 ± 6.2 years at baseline and 70.2 ± 6.2 years at the final follow-up. The mean follow-up period was 22.8 years.

Longitudinal changes [mean ± standard deviation (SD)] in radiographic variables from baseline to the final follow-up were as follows: LL from 48.1° ± 11.7° to 36.9° ± 17.6° (P < 0.001), SS from 35.6° ± 7.7° to 27.6° ± 11.9° (P < 0.001), PI from 55.4° ± 10.1° to 54.0° ± 12.0° (P = 0.238), PT from 19.9° ± 8.2° to 27.6° ± 11.8° (P < 0.001), SVA from 10.4 ± 27.9 to 25.8 ± 42.5 mm (P < 0.001), PI-LL from 7.4° ± 11.8° to 17.1° ± 18.2° (P < 0.001), FTA from 175.8° ± 2.5° to 177.1° ± 3.7° (P = 0.019), HKA from 183.4° ± 2.7° to 184.4° ± 4.0° (P = 0.029), mLDFA from 89.9° ± 2.7° to 87.9° ± 2.4° (P = 0.004), mMPTA from 91.1° ± 3.0° to 85.2° ± 2.4° (P < 0.001), and mLDTA from 92.5° ± 3.4° to 88.7° ± 3.2° (P < 0.001) (Table 1).

**Table 1:** Radiographic measurements at baseline and final follow-up in the 58 female volunteers.

Variable	Baseline, mean (SD)	Final follow-up, mean (SD)	P
Age (years)	47.4 (6.2)	70.2 (6.3)	< 0.001
Lumbar lordosis (°)	48.1 (11.7)	36.9 (17.6)	< 0.001
Sacral slope (°)	35.6 (7.7)	27.6 (11.9)	< 0.001
Pelvic incidence (°)	55.4 (10.1)	54.0 (12.0)	0.238
Pelvic tilt (°)	19.9 (8.2)	27.6 (11.8)	< 0.001
Sagittal vertical axis (mm)	10.4 (27.9)	25.8 (42.5)	0.003
Pelvic incidence minus Lumbar lordosis (°)	7.4 (11.8)	17.1 (18.2)	< 0.001
FTA (°)	175.8 (2.5)	177.1 (3.7)	0.019
HKA (°)	183.4 (2.7)	184.4 (4.0)	0.029
mLDFA (°)	89.9 (2.7)	87.9 (2.4)	< 0.001
mMPTA (°)	91.1 (3.0)	85.2 (2.4)	< 0.001
mLDTA (°)	92.5 (3.4)	88.7 (3.2)	< 0.001

*SD* standard deviation, *FTA* femorotibial angle, *HKA* hip–knee–ankle angle, *mLDFA* mechanical lateral distal femoral angle, *mMPTA* medial proximal tibia angle, *mLDTA* mechanical lateral distal tibia angle.

**Figure 3:**
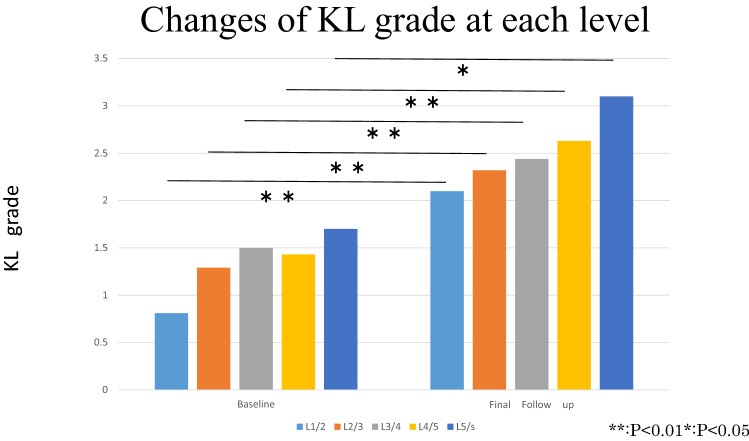
Lumbar arthritis was measured using the Kellgren–Lawrence grade at baseline and follow up. All Kellgren–Lawrence grades for the lumbar spine were increased with a significant difference. The L5/S had the highest grade at both, baseline and follow up.

Figure 3 shows changes in KL grade from baseline to final follow-up in both the knee joint and lumbar spine at each level. K1/2 changed from 0.8 to 2.1 (P < 0.001), K2/3 from 1.2 to 2.3 (P < 0.001), L3/4 from 1.4 to 2.4 (P < 0.001), L4/5 from 1.4 to 2.6 (P < 0.001), and L5/S from 1.7 to 3.1 (P = 0.021). The mean KL grade of the knee joint changed from 0.8 to 1.7 (P < 0.001).

Statistical analyses revealed significant longitudinal changes in all parameters except for PI. The correlation coefficients between changes in sagittal spinal alignment parameters and changes in coronal lower extremities alignment parameters are summarized in Table 2. Changes in PI-LL and changes in FTA showed the strongest correlation.

**Table 2:** Correlations between changes in spinal sagittal and coronal lower extremity parameters.

	∆FTA	∆HKA	∆mLDFA	∆mMPTA	∆mLDTA
∆LL	− 0.404** (P < 0.001)	− 0.287* (P = 0.029)	− 0.181 (P = 0.174)	0.109 (P = 0.416)	0.258 (P = 0.05)
∆SS	− 0.221 (P = 0.095)	− 0.168 (P = 0.208)	− 0.176 (P = 0.187)	0.048 (P = 0.723)	0.22 (P = 0.097)
∆PI	0.095 (P = 0.477)	0.212 (P = 0.109)	− 0.113 (P = 0.397)	0.128 (P = 0.339)	0.053 (P = 0.691)
∆PT	0.205 (P = 0.124)	0.308* (P = 0.019)	0.004 (P = 0.978)	0.14 (P = 0.295)	− 0.18 (P = 0.176)
∆SVA	0.303* (P = 0.021)	0.159 (P = 0.234)	0.03 (P = 0.822)	0.196 (P = 0.14)	0.078 (P = 0.56)
∆PI-LL	0.449** (P < 0.001)	0.412** (P < 0.001)	0.056 (P = 0.677)	0.006 (P = 0.961	− 0.243 (P = 0.066)

*LL* lumbar lordosis, *SS* sacral slope, *PI* pelvic incidence, *PT* pelvic tilt, *SVA* sagittal vertical axis, *FTA* femorotibial angle, *HKA* hip–knee–ankle angle, *mLDFA* mechanical lateral distal femoral angle, *mMPTA* mechanical medial proximal tibial angle, *mLDTA* mechanical lateral distal tibial angle.

**P < 0.01, *P < 0.05.

**Figure 4:**
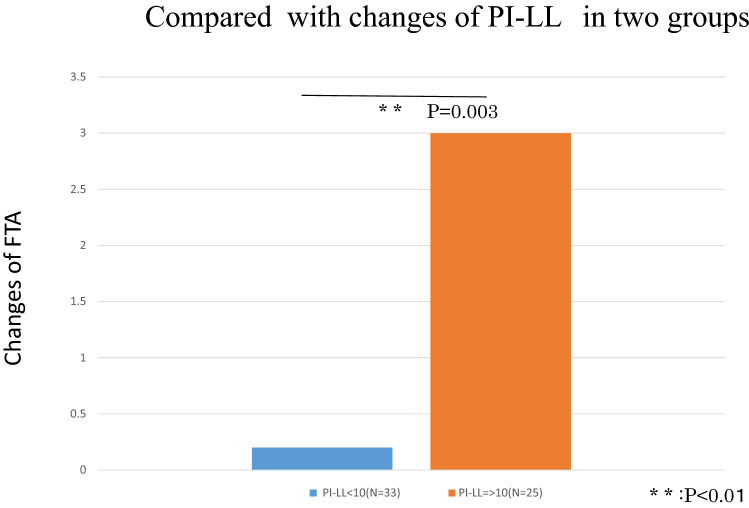
Comparison of changes in femorotibial angle (FTA) parameters among the two groups according to small or larger changes in PI-LL. The small group (PI-LL < 10) demonstrated significantly smaller changes in FTA.

When we divided volunteers into two groups based on changes in PI-LL, the L group showed a greater change in FTA compared to the S group (Fig. 4). The correlation coefficients between changes in lumbar spondylosis and changes in KOA are summarized in Table 3. Changes in knee KL grade and changes in L3/4 lumbar spondylosis showed the strongest correlation.

**Table 3:** Correlations between changes in KL grade and knee joint and lumbar spondylosis.

	∆L1/2	∆L2/3	∆L3/4	∆L4/5	∆L5/S
∆Knee joint	− 0.004 (P = 0.98)	0.118 (P = 0.39)	0.383** (P = 0.004)	0.333* (P = 0.012)	0.126 (P = 0.354)

*KL* Kellgren–Lawrence.

**P < 0.01, *P < 0.05.

All ICC values for both, inter- and intra-rater reliability for spinal parameters were good to excellent, as summarized in Table 4.

**Table 4:** Inter- and intra-observer reliability of spinal parameters.

Reliability	ICC	95% CI
**Intraobserver**		
LL	0.98	0.98–0.97
SS	0.99	0.98–0.99
PI	0.98	0.98–0.99
PT	0.98	0.97–0.99
SVA	0.99	0.98–0.99
PI-LL	0.98	0.97–0.99
**Interobserver**		
LL	0.91	0.74–0.97
SS	0.84	0.57–0.94
PI	0.81	0.50–0.93
PT	0.85	0.60–0.94
SVA	0.73	0.37–0.90
PI-LL	0.85	0.58–0.95

## Discussion

In this study, changes in PI-LL were related to changes in FTA over the period of this study (over 22 years). Changes in lumbar spondylosis were related to changes in KOA grade. These results indicated that progression of adult spinal deformity and progression of KOA occur in tandem.

In our study, PI did not change significantly over 22 years. PI has been reported as an individual anatomic parameter which is not affected by posture and remains unchangeable after growth^12–14^. Furthermore, PI becomes stable around 10 years of age, and varies widely from 33° to 85°^15,16^. Pelvic incidence does not change with age^17^. Spinal parameters over 22 years showed a decrease in LL and an increase in PT and SVA, respectively, which is similar to the changes reported in the previous reports of Uehara and Oe, which were studied in each age group^18,19^.

The prevalence of both lumbar pain and knee pain was examined epidemiologically by Yoshimura et al.^1^. In their large population-based cohort study of 9,046 participants, both lumbar pain and knee pain existed in 12.2% of participants, indicating that many patients suffered from both lumbar pain and knee pain.

With regard to clinical symptoms, Suri et al. reported that 57.4% of participants with symptomatic tibiofemoral KOA had LBP, and LBP was significantly related to increased Western Ontario and McMaster Universities (WOMAC) knee pain score^20^. Moreover, chronic LBP has been found to be a risk factor for poor outcomes following total knee replacement surgery^21^. Low back pain was most prevalent in non-surgically-treated knee joints before total knee replacement surgery^22^.

Barry et al. reported that compensatory mechanisms occur in the spine, pelvis, knee joints, and ankle joints to maintain the balance of the trunk. In major spinal deformity, flexion of the knee has been reported as the main compensatory mechanism for lack of LL^6^. When the International Spine Study Group examined the role of the lower extremities in compensation for spinal deformity, they found a correlation between spino-pelvic parameters and lower extremity parameters, which was useful for the understanding of the changes in the lower extremities^23–26^. Furthermore, Cheng et al. and Arima et al. indicated that the spontaneous change in knee flexion angle was significantly improved after surgical correction of ASD, which was demonstrated as a compensatory mechanism when ASD was progressive^27,28^.

In contrast to the spine affecting the knee joints, some studies have explored the knee–spine syndrome and examined the effect of the knees on spinal deformity. The effect of knee flexion on sagittal spinal alignment has been investigated by Lee et al. who asked 30 young men to wear a knee brace with extension restrictions to produce a knee flexion contracture model^29^. They found that with 15° and 30° of knee flexion, the participants showed a significantly reduced LL angle and a more forward inclination of the spine^29^. Moreover, Wang et al. examined sagittal alignment of the spine-pelvis-lower extremities in KOA patients, and found that severe KOA was related to hip flexion, decreased lumbar lordosis, and increased SVA^30^. Both the spine and knee eventually lead to the development of an anterior trunk bending position during standing and walking.

Generally, adjacent parameters have a higher correlation, but ΔFTA and ΔLL are correlated, whereas ΔFTA and ΔPT are not related in our study.

A decrease in lordosis in the lumbar spine produces a compensatory effect on the posterior tilting of the adjacent pelvis. In the case of OA of the knee, the progression of restriction of knee extension is not compensated for by the pelvis, but by the anterior tilt of the trunk. We speculate that the FTA was, therefore, correlated with LL and not PT. However, it is also possible that this could purely be related to knee and lumbar degeneration.

While many studies of the relationship between spinal sagittal alignment and sagittal lower extremity alignment have been reported, few reports have examined the relationship between spinal sagittal alignment and coronal lower extremity alignment.

Koga reported that varus deformity of the knee joint was affected by limited knee extension, and range of motion of the knee joint was changed to an eccentric movement. Furthermore, KOA progression occurred with three-dimensional changes including axial rotation^31^. With aging, the lower extremities change in the sagittal phase and coronal phase, and we speculate that flexion contracture and knee varus deformity occur in many populations.

In our study, there is a moderate correlation between ΔLL and ΔFTA, but the correlation between ΔLL and ΔHKA is low. In terms of this result, we speculate that there was a higher correlation between reduction in lumbar lordosis and the FTA using the femoral trunk axis, than with the HKA using the center of the femoral head.

In lower extremity parameters, FTA is one of the important global parameters used for surgical planning of total knee arthroplasty and osteotomy surgery. High tibial osteotomy (HTO) is a widely-accepted surgery for treatment of medial compartment KOA. Following this surgery, FTA remains an index of good long-term surgical results^32,33^. Kim et al. indicated that HTO improved SSA and spinal parameters significantly closer to that of healthy young subjects using surface body markers, and reduced abnormalities that may result in spinal problems, such as degeneration or pain^34^. Koshino et al. reported that 1° of change in knee flexion was equal to about 0.1° of change in FTA. Their study involved 17 young male volunteers and not elderly patients with KOA. However, we the relationship between knee flexion and changes in FTA should be considered in the assessment of knee–spine relationships^35^. Harato et al. reported that knee flexion contracture of severe KOA leads to three-dimensional changes in trunk motions and KOA leads to spinal sagittal malalignment of the lumbar spine^36^. Our study showed that the relationship between changes in SSA parameters and changes in coronal lower extremity alignment parameters were important factors to consider in the longitudinal changes in the spine and lower extremities.

We found that changes in KOA and changes in lumbar spondylosis at L3/4 and L4/5 were strongly correlated. Muraki et al. reported that lumbar spondylosis with KL ≥ 3 occurred in L3/4 and L4/5 in women in a large population-based cohort study^37^. Moreover, Akeda et al. and Hassett et al. reported that the presence of KOA was significantly associated with a risk of the development of lumbar disc height narrowing in the elderly population^38,39^. It was very difficult to determine which factor was the primary factor influencing the lumbar spondylosis changes or KOA in our study, and our results suggest that both the lumbar joints and the knee joints affected each other.

We could not determine whether spinal and lower extremity changes were compensatory or merely concurrent with age-related changes; however, it is necessary to evaluate the state of the lower extremity joints for successful management of ASD.

This study had several limitations. Firstly, the number of volunteers in our study was small, so the recorded changes in parameters may have been limited. Secondly, sagittal lower extremities were not evaluated in our study. However, in a previous study, it was demonstrated that adult spinal deformity affected sagittal lower extremity alignment^6^. Thirdly, our study only included female volunteers, and a different result may have occurred if we included male volunteers due to sex differences in muscle, bone, cartilage, and discs. Fourth, we were not able to assess long-term longitudinal changes with respect to the symptoms. This warrants further study.

## Conclusions

In conclusion, we showed that changes in PI-LL showed the strongest correlation with changes in FTA, which is one of the global coronal joint alignment parameters used for KOA evaluation. Over 22 years, changes in lumbar spondylosis at L3/4 and L4/5 were related to changes in KOA. In successful management of ASD, it is necessary to evaluate the state of the lower extremity alignment, not only in the sagittal phase, but also the coronal phase.
